# Photoluminescence processes in *τ*-phase Ba_1.3_Ca_0.7-*x*-*y*_SiO_4_:*x*D*y*^3+^/*y*Tb^3+^ phosphors for solid-state lighting

**DOI:** 10.1098/rsos.220101

**Published:** 2022-06-15

**Authors:** Desta R. Golja, Menberu M. Woldemariam, Francis B. Dejene, Jung Yong Kim

**Affiliations:** ^1^ Department of Materials Science and Engineering, Jimma Institute of Technology, Jimma University, P.O. Box 378, Jimma, Ethiopia; ^2^ Department of Physics, Jimma University, P.O. Box 378, Jimma, Ethiopia; ^3^ Department of Physics, University of the Free State (QwaQwa Campus), P.O. Box 339, Bloemfontein, South Africa; ^4^ Department of Chemical and Physical Sciences, Walter Sisulu University (Mthatha Campus), Private Bag XI UNITRA 5117, South Africa; ^5^ Department of Materials Science and Engineering, Adama Science and Technology University, P.O. Box 1888, Adama, Ethiopia; ^6^ Center of Advanced Materials Science and Engineering, Adama Science and Technology University, P.O. Box 1888, Adama, Ethiopia

**Keywords:** phosphors, rare-earth ion, silicate, light-emitting diode, structure–property relation, photoluminescence

## Abstract

The *τ*-phase Ba_1.3_Ca_0.7-*x*-*y*_SiO_4_:*x*D*y*^3+^/*y*Tb^3+^ phosphors co-doped with D*y*^3+^ (*x* = 0.03) and Tb^3+^ (*y* = 0.01–0.05) trivalent rare-earth ions were prepared by the gel-combustion method. The structure–property relation of the samples was examined by X-ray diffraction, scanning electron microscopy and spectrophotometer. Here, the effect of Tb^3+^'s concentration on the spectroscopic properties of Ba_1.3_Ca_0.7-*x*-*y*_SiO_4_:*x*D*y*^3+^/*y*Tb^3+^ phosphors was explored by using the photoluminescence excitation, emission and decay curves. Importantly, the photonic energy transfer from (D*y*^3+^:^4^F_9/2_ + Tb^3+^:^7^F_6_) to (D*y*^3+^:^6^H_15/2_ + Tb^3+^:^5^D_4_) was observed, in which the D*y*^3+^ ions act as a light-emitting donor whereas the Tb^3+^ ions as a light-absorbing acceptor, resulting in an enhanced emission from the co-doped Ba_1.3_Ca_0.7-*x*-*y*_SiO_4_:*x*D*y*^3+^/*y*Tb^3+^ (*x* = 0.03 and *y* = 0.01–0.05) phosphors. Finally, the chromaticity coordinates were determined from the measured emission spectra, locating at the green and white light regions. This observation indicates that the characteristic emission colour could be tuned from white to green by varying Tb^3+^ concentrations under ultraviolet light.

## 1. Introduction

The phosphor materials have been used as a semiconductor for a next-generation lighting device, e.g. lasers and light-emitting diodes (LEDs) [1,2]. Among them, the alkaline earth silicates doped with a rare-earth element are promising for versatile photonic materials and devices such as a white-LED, long-lasting and multi-colour phosphors, silicon solar cells and others [3–7]. Specifically, the trivalent rare-earth (RE^3+^) ions exhibit a characteristic intra-4f shell luminescence, enabling them somewhat free from the choice of host materials [8–10]. However, in spite of this independence, the polycrystalline silicates doped with alkaline earth atoms (Ca and Ba) have been widely used as a host matrix due to the apparent advantages such as simple synthesis, durability, structural stability, resistance to chemical change, easy thermal preparation and strong absorption properties in the near-ultraviolet (UV) region [11,12]. In general, the RE^3+^ ions have abundant electronic structures, leading to various energy emissions under the excitation of appropriate UV light. For example, the dysprosium ion (Dy^3+^)-doped hosts exhibit characteristic intense visible emission bands at 470–500 nm (blue), 570–600 nm (yellow) and weak 645–665 nm (red), whereas Tb^3+^ displays an intense green luminescence at approximately 545 nm [13]. Furthermore, when D*y*^3+^ ions were incorporated into a silicate phosphor, they could serve as a blue-light-absorbing activator for the white-LED applications through the excitation by a near UV-blue light. Hence, D*y*^3+^ ions are a good sensitizer by which a partial energy transfer is available to other activator ions such as the terbium ions (Tb^3+^) [14]. Specifically, the energy in the ^4^F_9/2_ level of D*y*^3+^ could be transferred to the ^5^D_4_ level of Tb^3+^ by the energetic resonance. Then, the population of Tb^3+^'s ^5^D_4_ level should be increased, resulting in enhanced green luminescence. Here note that the cross-relaxation between D*y*^3+^ and Tb^3+^ is known to be another origin for the enhancement of population in the aforementioned level [14,15]. To this end, the dopant/co-dopant engineering should be useful when its concentrations are optimized.

In this work, we report on the photoluminescence (PL) processes in the polycrystalline ceramic phosphor (Ba_1.3_Ca_0.7-*x*-*y*_SiO_4_:*x*D*y*^3+^/*y*Tb^3+^) based on the crystal structure–property relationship. Herein, the above silicate phosphor was chosen as a host material because of its special *τ*-phase crystal structure, excellent chemical stability and appropriate energy bandgap (approx. 3.2 eV) [16–18]. Note that the single-ion doping effects on this silicate phosphor were reported in our previous works [17,18]. Hence, considering the importance of white-LED development [19–21], the D*y*^3+^/Tb^3+^'s co-doping effect on the structural, morphological, photoluminescent and other properties of Ba_1.3_Ca_0.7_SiO_4_ phosphor were investigated for elucidating the PL processes in *τ*-phase co-doped Ba_1.3_Ca_0.7-*x*-*y*_SiO_4_:*x*D*y*^3+^/*y*Tb^3+^ phosphors, suggesting that these ceramic phosphors are a promising candidate for white-LED and/or laser applications.

## 2. Methods

### 2.1. Preparation of phosphor materials

Ba_1.3_Ca_0.7-*x*-*y*_SiO_4_:*x*D*y*^3+^/*y*Tb^3+^ (*x* = 0.03 and *y* = 0.01–0.05) phosphors were prepared by using a gel-combustion method. Here, it is notable that the concentration of D*y*^3+^ ions was fixed as 3 mol% based on our preliminary experiments, showing the highest performant at this composition when tested in the range of 1–5 mol% D*y*^3+^ ions [22]. The raw materials used in the preparation process were Ba(NO_3_)_2_ (99.9%), Ca(NO_3_)_2_.4H_2_O (99.9%), D*y*(NO_3_)_3_ (99.9%), Tb(NO_3_)_3_ (99.9%), CH_4_N_2_O (99.9%) and Si(OC_2_H_5_)_4_ (99.9%). In order to introduce D*y*^3+^/Tb^3+^ co-dopant ions into the Ba_1.3_Ca_0.7_SiO_4_ host, 3.0 mol% of D*y*^3+^ and 1–5 mol% of Tb^3+^ have been selected. Stoichiometric amounts of Ba(NO_3_)_2_, Ca(NO_3_)_2_.4H_2_O and CH_4_N_2_O were dissolved in 10 ml of deionized water [17,18]. The estimated quantities of D*y*^3+^ and Tb^3+^ ions were added to the solution as doping and co-doping ions. Then, the required amount of tetraethyl orthosilicate (TEOS) was dissolved into 10 ml of ethanol and added dropwise to the above solution while vigorously stirring. The mixtures were continuously stirred at approximately 80°C until the transparent solution was formed. Finally, the transparent solution was quickly transferred to alumina crucible and put into a preheated furnace at approximately 550°C. The whole process took 5–10 min. Polycrystalline Ba_1.3_Ca_0.7-*x*-*y*_SiO_4_:*x*D*y*^3+^/*y*Tb^3+^ (*x* = 0.03 and *y* = 0.01–0.05) powders obtained after annealing the samples at 1000°C for 2 h were then crushed to fine powders for further characterization. Note that for preparing the aforementioned phosphors, the regent-grade chemicals such as barium nitrate Ba(NO_3_)_2_, calcium nitrate tetrahydrate Ca(NO_3_)_2_·4H_2_O, TEOS Si(OC_2_H_5_)_4_, urea CH_4_N_2_O and others were purchased from Sigma-Aldrich Chemical Co.

### 2.2. Characterization

X-ray diffraction (XRD) measurements (model Philips Bruker D8 advance) were carried out to examine the structure and phase of the prepared phosphor materials. The scanning electron microscope (SEM) (model JEOL JSM-7800F) was used to characterize the morphology of phosphors as a function of composition. Energy-dispersive X-ray spectroscope (EDX) (Oxford Aztec) was used to confirm the composition of elements in the Ba_1.3_Ca_0.7-*x*-*y*_SiO_4_:*x*D*y*^3+^/*y*Tb^3+^ (*x* = 0.03 and *y* = 0.01–0.05) samples. The PL emission and decay spectra were investigated using the Cary Eclipse fluorescence spectrophotometer (model LS-55) equipped with a xenon flash lamp to act as the excitation source.

## 3. Results and discussion

### 3.1. Crystal structure analysis

**Figure 1.**
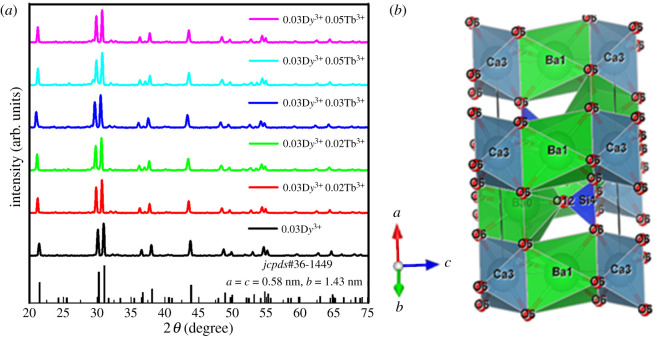
(*a*) XRD patterns of Ba_1.3_Ca_0.7-*x*-*y*_SiO_4_:*x*D*y*^3+^/*y*Tb^3+^ (*x* = 0.03 and *y* = 0.00–0.05) crystals. (*b*) Crystal structure of Ba_1.3_Ca_0.7_SiO_4_ (host lattice) based on the VESTA software.

Figure 1*a* shows the XRD diffraction patterns of Ba_1.3_Ca_0.7-*x*-*y*_SiO_4_:*x*D*y*^3+^/*y*Tb^3+^ (*x* = 0.03 and *y* = 0.01–0.05) with the corresponding standard data file (JCPDS card no. 36-1449). Here, there is no discernable peak related with impurities, indicating that the co-dopants (D*y*^3+^/Tb^3+^) are appropriately incorporated into the hexagonal Ba_1.3_Ca_0.7_SiO_4_ phosphors with space group (p$\bar{3}$m1) [16,17,23]. Figure 1*b* shows the crystal structure of alkaline earth-based tetra silicate, Ba_1.3_Ca_0.7_SiO_4_, constructed by using the VESTA (*visualization for electronic structural analysis*) software. The crystal structure of Ba_1.3_Ca_0.7_SiO_4_ has the hexa-coordinated Ba/Ca sites and the tetrahedral anion group ${\mathrm{SiO}}_{4}^{4-}$ [24]. Thus, the unit cell environment of Ba_1.3_Ca_0.7_SiO_4_ phosphors is similar to the Ba-O and Ca-O crystal coordination [18]. Importantly, the trivalent dopant ions D*y*^3+^ and Tb^3+^ may substitute Ca^2+^ ions instead of Ba^2+^ in the matrix Ba_1.3_Ca_0.7_SiO_4_ [16,25–27]. This is because the ionic radii of D*y*^3+^ (1.03 Å) and Tb^3+^ (1.18 Å) are similar to that of Ca^2+^ (1.06 Å) and different from Ba^2+^ (1.34 Å) [28–31]. Note that depending on substitutions, the crystal structure of the host could be stabilized or destabilized. Furthermore, the difference in ionic valency (D*y*^3+^/Tb^3+^ and Ca^2+^) may result in the uncompensated ions [32], which is the so-called ‘aliovalent effect’ and is considered as future work.

**Figure 2.**
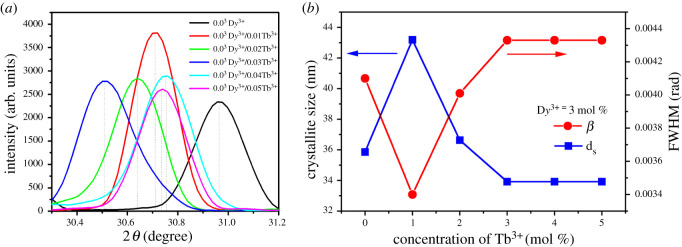
(*a*) Magnified view of the (110) XRD peaks. (*b*) Crystallite size and FWHM as a function of Tb^3+^ concentration for the co-doped Ba_1.3_Ca_0.7-*x*-*y*_SiO_4_:*x*D*y*^3+^/*y*Tb^3+^ (*x* = 0.03 and *y* = 0.00–0.05) phosphors.

The Gaussian fits of (110) diffraction peaks are shown in figure 2*a*, displaying the variation of interplanar *d*-spacing (0.2918 ± 0.0016 nm) depending on the co-dopant Tb^3+^ concentration. The insertion of the dopant atoms into the host lattice may bring forth a subtle change in the crystal structure. This modification could be detected through the peak shift and/or the intensity change in the XRD patterns. Here, the shift of peak originated from the different atomic sizes (or ionic radii), dopant concentration and interactions among constituent atoms in the condensed phase, bringing forth an expansion or contraction of the lattice parameter. Hence, the XRD peak could be shifted. Specifically, the shift to a lower angle indicates that the lattice is partially expanded. Note that the ionic radius (1.03 Å) of D*y*^3+^ is very close to that (1.06 Å) of Ca^2+^. However, in the case of Tb^3+^, its ionic radius (1.18 Å) is larger than that of Ca^2+^, indicating that after Tb^3+^ doping, the lattice may receive more internal stress. Furthermore, the uncompensated valency might be a factor in this shift because internal interaction between constitutes should be changed. However, this internal stress should be within a tolerance level because the crystal structure of phosphors retains its original *τ*-phase structure according to XRD patterns in figure 1*a*. On the other hand, the change of peak intensity is related to the crystallite size as well as the electronic environment in the presence of dopant ions. Indeed, when compared with the single-doped phosphor, the co-doped ones exhibited a diffraction peak at slightly lower angles as a function of Tb^3+^ concentration (figure 2*a*). In addition, the peak broadening was also observed from which the crystallite size can be estimated (figure 2*b*). At this moment, it is noteworthy that because D*y*^3+^, Tb^3+^ and Ca^2+^ have the same six coordination numbers, the dopant ions are expected to partially substitute the host Ca^2+^ sites as noted before. Otherwise, the dopants could stay in vacant or interstitial sites as well as in defects (e.g. grain boundaries). Furthermore, the volume (*V*) of the unit cells was expanded from 454.72 Å^3^ to 481.53 ± 13.78 Å^3^ when the D*y*^3+^ single-doped phosphor was co-doped additionally with Tb^3+^ ions (table 1) [33–35].

**Table 1.** The crystallite size ($D_{S}$) and lattice parameter (a, c and unit cell volume) for the co-doped Ba_1.3_Ca_0.7-*x*-*y*_SiO_4_:*x*D*y*^3+^/*y*Tb^3+^ (*x* = 0.03 and *y* = 0.00–0.05) phosphors.

0.03D*y*^3+^/*y*Tb^3+^(*y* = 0.00–0.05)	FWHM	angle	crystallite size	lattice parameter		
	β (radian)	θ (degree)	$D_{S}$ (nm)	a (Å)	c (Å)	volume (Å^3^)
*y* = 0.00	0.00410	15.48	35.86	5.60	14.50	454.72
*y* = 0.01	0.00340	15.26	43.19	5.70	14.72	478.25
*y* = 0.02	0.00401	15.32	36.63	5.65	14.50	462.87
*y* = 0.03	0.00433	15.26	33.92	5.71	14.65	477.65
*y* = 0.04	0.00433	15.28	33.92	5.75	14.80	489.33
*y* = 0.05	0.00433	15.27	33.92	5.80	14.85	499.55

Figure 2*b* shows the crystallite size and the full width at half maximum (FWHM) as a function of the co-dopant Tb^3+^ concentration, displaying that the FWHM decreases at 1 mol% Tb^3+^ and subsequently increases and then saturates at 3–5 mol% Tb^3+^. Here, the average crystallite size ($D_{S}$) can be calculated based on Scherrer's equation,

3.1 $$D_{S}=\frac{0.92\lambda }{\beta \cos\theta },$$

where λ (= 1.5406 Å) is the wavelength of the X-ray and β is the FWHM at angle θ (table 1). In figure 2*b*, the $D_{S}$ data should reverse the trend observed in β according to the relation of $D_{S}\propto 1/\beta$, suggesting that the structural morphologies of phosphors could be optimized for desirable optoelectronic properties through the doping processes based on the structure–property relationship of materials science.

### 3.2. Morphological and elemental analyses

**Figure 3.**
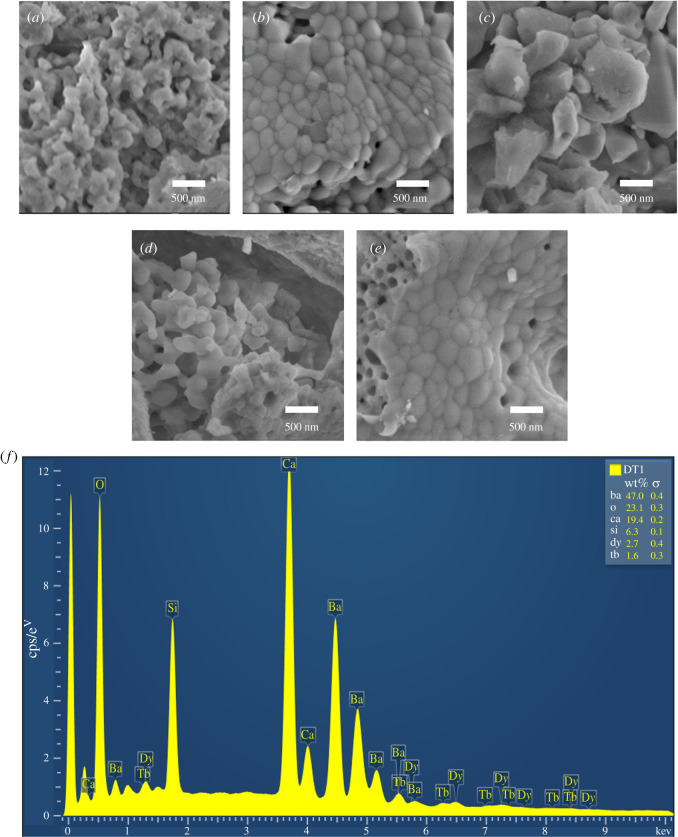
The SEM images: (*a*) Ba_1.3_Ca_0.7_SiO_4_ (host), (*b*) Ba_1.3_Ca_0.7-*x*-*y*_SiO_4_: *x*D*y*^3+^/*y*Tb^3+^ (*x* = 0.03 and *y* = 0.00), (*c*) Ba_1.3_Ca_0.7-*x*-*y*_SiO_4_: *x*D*y*^3+^/*y*Tb^3+^ (*x* = 0.03 and *y* = 0.01), (*d*) Ba_1.3_Ca_0.7-*x*-*y*_SiO_4_: *x*D*y*^3+^/*y*Tb^3+^ (*x* = 0.03 and *y* = 0.03), (*e*) Ba_1.3_Ca_0.7-*x*-*y*_SiO_4_: *x*D*y*^3+^/*y*Tb^3+^ (*x* = 0.03 and *y* = 0.05) and (*f*) the SEM-EDX analysis for Ba_1.3_Ca_0.7-*x*-*y*_SiO_4_: *x*D*y*^3+^/*y*Tb^3+^ (*x* = 0.03 and *y* = 0.01) phosphor.

Figure 3*a* shows the morphology of the host Ba_1.3_Ca_0.7_SiO_4_ phosphor, displaying an irregular microstructure. However, when this phosphor was single-doped with 3 mol% of D*y*^3+^ ion (i.e. of Ba_1.3_Ca_0.7-*x*_SiO_4_:*x*D*y*^3+^ where *x* = 0.03), the microstructural morphology changed dramatically, exhibiting relatively uniform grains and grain boundaries. This kind of uniformity might be useful for photonic device applications [36,37]. Furthermore, the trivalent rare-earth ion co-doped Ba_1.3_Ca_0.7-*x*-*y*_SiO_4_:*x*D*y*^3+^/yTb^3+^ (*x* = 0.03 and *y* = 0.01, 0.03 and 0.05) phosphors were also characterized for their morphologies (figure 3*c–e*). As shown in figure 3*c–e*, depending on the co-dopant Tb^3+^ concentration, the co-doped phosphors display versatile microstructures, which should affect the photonic and optoelectronic properties in solid-state lighting devices. Interestingly, in line with the XRD data in figure 2, the 1 mol% Tb^3+^ co-doped phosphor shows the largest polycrystalline domains among samples as shown in figure 2*c*. In general, the closely packed agglomeration of the phosphor particles is known to be helpful for fabricating high-performance lighting devices with the intense luminescence and small scattering [38,39]. Here, it is noteworthy that the sequence of particle/aggregation size does not necessarily match that of the crystallite size in figure 3*b* and table 1 because the latter is the average single-crystalline size in the former (i.e. a polycrystalline domain with partially disordered or amorphous regions). In figure 3*f*, the energy-dispersive X-ray spectroscopy (EDX) spectra exhibit the chemical composition of the D*y*^3+^/Tb^3+^ co-doped phosphor (Ba_1.3_Ca_0.7-*x*-*y*_SiO_4_:*x*D*y*^3+^/*y*Tb^3+^ where *x* = 0.03 and *y* = 0.01), confirming the presence of component elements including the two dopant atoms, i.e. D*y* and Tb.

### 3.3. Photoluminescence processes in D*y*^3+^/Tb^3+^ co-doped Ba_1.3_Ca_0.7_SiO_4_ phosphors

**Figure 4.**
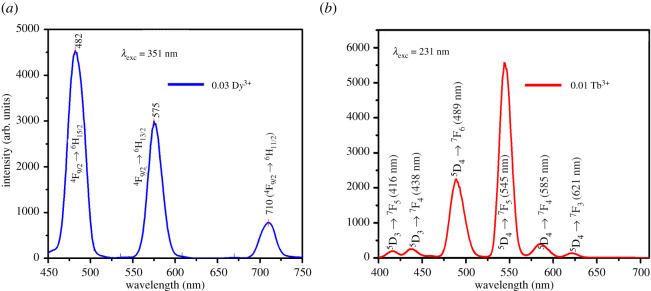
Emission spectra of (*a*) the single-doped Ba_1.3_Ca_0.7-*x*-*y*_SiO_4_:*x*D*y*^3+^/*y*Tb^3+^ (*x* = 0.03 and *y* = 0.00) at the excitation wavelength of $\lambda_{exc}=351\;\mathrm{nm}$ and (*b*) the other single-doped Ba_1.3_Ca_0.7-*x*-*y*_SiO_4_:*x*D*y*^3+^/*y*Tb^3+^ (*x* = 0.00 and *y* = 0.01) at the excitation wavelength of $\lambda_{exc}=231\;\mathrm{nm}$.

When the single-doped Ba_1.3_Ca_0.7-*x*-*y*_SiO_4_:*x*D*y*^3+^/*y*Tb^3+^ (*x* = 0.03 and *y* = 0.00) phosphor was excited at 351 nm [40], the emission peaks were clearly observed at 482, 575 and 710 nm, arising from the radiative emission transition from the F orbital to the various H orbitals (figure 4*a*). Specifically, the emission spectra at 482 nm (blue), 575 nm (yellow) and 710 nm (red) are attributed to ^4^F_9/2_ → ^6^H_15/2,_^4^F_9/2_ → ^6^H_13/2_ and ^4^F_9/2_ → ^6^H_11/2_ transitions, respectively. The emission peak at 482 nm originates from the magnetic dipole transitions, whereas that at 575 nm is ascribed to the electric dipole transition (i.e. a hypersensitive transition) [41,42]. According to Judd–Ofelt theory [40,43], the yellow emission during ^4^F_9/2_ → ^6^H_13/2_ transition will be dominant when the D*y*^3+^ ions are placed at low-symmetry sites without an inversion centre, while the blue emission during ^4^F_9/2_ → ^6^H_15/2_ transition is stronger when D*y*^3+^ is placed at high symmetry with an inversion centre.

Figure 4*b* shows the PL emission spectra of the other single-doped Ba_1.3_Ca_0.7-*x*-*y*_SiO_4_: *x*D*y*^3+^/*y*Tb^3+^ (*x* = 0.00 and *y* = 0.01) phosphor. The most prominent emission band at 545 nm corresponds to a bright green originating from the ^5^D_4_ → ^7^F_5_ transition, indicating the high probability of the electric dipole transition. The other transitions ^5^D_3_ or ^5^D_4_ to ^7^F*_J_* produce a wide coverage emission in the visible spectrum. The peaks at 416 and 438 nm are due to ^5^D_3_ → ^7^F*_J_* (*J* = 5, 4) transition, whereas the peaks at 489, 545, 585 and 621 nm are ascribed to 4f → 4f transitions from ^5^D_4_ → ^7^F*_J_* (*J* = 6, 5, 4, 3), respectively.

**Figure 5.**
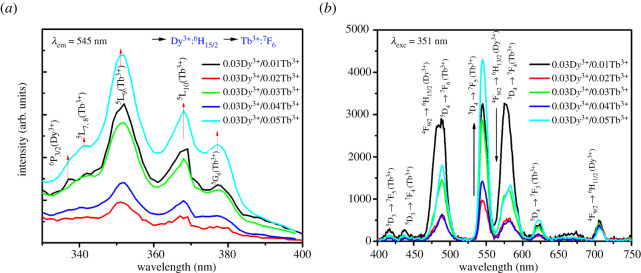
(*a*) Excitation and (*b*) emission spectra of co-doped Ba_1.3_Ca_0.7-*x*-*y*_SiO_4_:*x*D*y*^3+^/*y*Tb^3+^ (*x* = 0.03 and *y* = 0.01–0.05) phosphors, when the emission wavelength ($\lambda_{\mathrm{em}}$) is 545 nm and the excitation wavelength ($\lambda_{\mathrm{exc}}$) is 351 nm, respectively.

The PL processes of the co-doped Ba_1.3_Ca_0.7-*x*-*y*_SiO_4_: *x*D*y*^3+^/*y*Tb^3+^ (*x* = 0.03 and *y* = 0.01–0.05) phosphors were examined by excitation and emission spectroscopy. As shown in figure 5*a*, the spectrum shows the five excitation bands including the four excitation bands of Tb^3+^ ions (^7^F_6_ → ^5^L_7,8_ (341 nm), ^7^F_6_ → ^5^L_9_ (351 nm), ^7^F_6_ → ^5^L_10_ (370 nm) and ^7^F_6_ → ^5^G_6_ (378 nm) transitions) and one excitation bands of D*y*^3+^ ions (^6^H_15/2_ → ^6^P_3/2_ (337 nm) transition) [44].

Figure 5*b* displays the emission spectra of the Ba_1.3_Ca_0.7-*x*-*y*_SiO_4_:*x*D*y*^3+^/*y*Tb^3+^ (*x* = 0.03 and *y* = 0.01–0.05) phosphors at the excitation wavelength of 351 nm. As shown in figure 5*b*, the emission spectra exhibit mainly nine emission bands due to the combined f ↔ f electronic transition of D*y*^3+^ and Tb^3+^ ions as expected from each single-doped phosphors in figure 4*a,b*. Three emission bands are ascribed to D*y*^3+^ ions of ^4^F_9/2_ → ^6^H_15/2_ (blue, 482 nm), ^4^F_9/2_ → ^6^H_13/2_ (yellow, 575 nm) and ^4^F_9/2_ → ^6^H_11/2_ (red, 707 nm) and the other six emission bands at 416, 438, 489, 545, 585 and 621 nm are corresponding to ^5^D_3_ → ^7^F_5_, ^5^D_3_ → ^7^F_4_, ^5^D_4_ → ^7^F_6_, ^5^D_4_ → ^7^F_5_, ^5^D_4_ → ^7^F_4_ and ^5^D_4_ → ^7^F_3_ transitions of Tb^3+^ ions in the phosphor compounds, respectively [45,46]. In this work, the location of the assigned bands was calibrated by the emission wavelength of commercial InGaN UV-LED. Accordingly, the excitation wavelength at 351 nm is appropriate for studying the photoluminescent process (e.g. white or green light emission) in Ba_1.3_Ca_0.7-*x*-*y*_SiO_4_:*x*D*y*^3+^/*y*Tb^3+^ (*x* = 0.03 and *y* = 0.01–0.05) phosphors [44,46]. Furthermore, it was observed that after co-doping, the decay time of phosphors was extended (table 2). This phenomenon may be ascribed to the energy transfer between D*y*^3+^ and Tb^3+^ ions (and/or to the re-absorption of D*y*^3+^'s emission light by Tb^3+^ ions): D*y*^3+^ (^4^F_9/2_) + Tb^3+^ (^7^F_6_) → D*y*^3+^ (^6^H_15/2_) + Tb^3+^ (^5^D_4_).

**Table 2.** The decay time parameters for Ba_1.3_Ca_0.7-*x*-*y*_SiO_4_:*x*D*y*^3+^/*y*Tb^3+^ (*x* = 0.03 and *y* = 0.00–0.05) phosphors. The physical parameters $\tau_{m}$ and $\tau_{\;fit}$ are the decay time from measurement and fitting, respectively; $R_{0}$ is the critical transfer distance, and $\eta_{ET}$ is the energy transfer efficiency.

phosphors Ba_1.3_Ca_0.7-*x*-*y*_SiO_4_: *x*D*y*^3+^/*y*Tb^3+^ (*x* = 0.03 and *y* = 0.00–0.05)	$\tau_{m}$ (ms)	$\tau_{\;fit}$ (ms)	$R_{0}$ (Å)	$\eta_{ET}$ (%)	CIE coordinates (*x*, *y*)
*y* = 0.00	2.1	—	—	—	(0.288, 0.338)
*y* = 0.01	3.4	3.1	5.7	66.8	(0.346, 0.449)
*y* = 0.02	3.1	3.2	6.8	33.0	(0.338, 0.453)
*y* = 0.03	3.0	2.9	6.7	66.0	(0.339, 0.497)
*y* = 0.04	3.1	2.6	6.2	28.0	(0.336, 0.498)
*y* = 0.05	3.0	2.2	5.6	33.8	(0.337, 0.520)

For the 0.01Tb^3+^ single-doped sample in figure 4*b*, the most intense peak is at 545 nm (^5^D_4_ → ^7^F_5_), the second intense one at 489 nm (^5^D_4_ → ^7^F_6_), and the third intense one at 585 nm (^5^D_4_ → ^7^F_4_). However, for co-doped samples, as shown in figure 5*b*, the relative intensity was changed as a function of Tb^3+^ concentration. For example, in the co-doped 0.03D*y*^3+^/0.01Tb^3+^ condition, both the peaks at 489 nm (^5^D_4_ → ^7^F_6_) and 585 nm (^5^D_4_ → 7F_4_) were intensified compared with the most intense peak at 545 nm (^5^D_4_ → ^7^F_5_). Second, at the 0.03D*y*^3+^/(0.02–0.05)Tb^3+^ conditions, the peak at 585 nm (^5^D_4_ → ^7^F_4_) was relatively intensified when compared with the most intense peak at 545 nm (^5^D_4_ → ^7^F_5_). Therefore, when co-doped, the green light at 545 nm (^5^D_4_ → ^7^F_5_) was commonly intensified because of energy transfer from D*y*^3+^ to Tb^3+^ ions. Here, the efficiency of energy transfer ($\eta_{ET}$) from D*y*^3+^ to Tb^3+^ ions in the emission spectra (figure 5*b*) could be explained using the below formula [47],

3.2 $$\eta_{ET}=1-\frac{I_{Dy^{3+}}}{I_{Dy^{3+}}+I_{Tb^{3+}}}$$

where $I_{Dy^{3+}}$ and $I_{Tb^{3+}}$ are the integrated intensities of 575 nm (D*y*^3+^) and 545 nm (Tb^3+^) emission bands corresponding to ^4^F_9/2_→^6^H_13/2_ and ^5^D_4_→^7^F_5_ transitions, respectively. The $\eta_{ET}$ values were summarized in table 2. It was investigated that the maximum $\eta_{ET}$ is 66.8% at the co-doped Ba_1.3_Ca_0.7-*x*-*y*_SiO_4_:*x*D*y*^3+^/*y*Tb^3+^ (*x* = 0.03 and *y* = 0.01) samples, indicating that if one would like to use the green light emission (^5^D_4_ → ^7^F_5_ at 545 nm) through Tb^3+^ ions, 1 mol% Tb^3+^ could be a promising condition in the presence of 3 mol% D*y*^3+^ ions to synthesize the co-doped Ba_1.3_Ca_0.7-*x*-*y*_SiO_4_:*x*D*y*^3+^/*y*Tb^3+^ (*x* = 0.03 and *y* = 0.01–0.05) phosphors. Notably, Huerta *et al*. characterized the lithium-aluminium-zinc phosphate glass co-doped with D*y*^3+^/Tb^3+^, in which the D*y*^3+^ emission decay time increment in the presence of Tb^3+^ has been attributed to changes in the phonon energies of the host, resulting in a decrease in the non-radiative decay rate of the dysprosium ^4^F_9/2_ emitting level that could compete with the D*y*^3+^ to Tb^3+^ non-radiative energy transfer rate [48].

**Figure 6.**
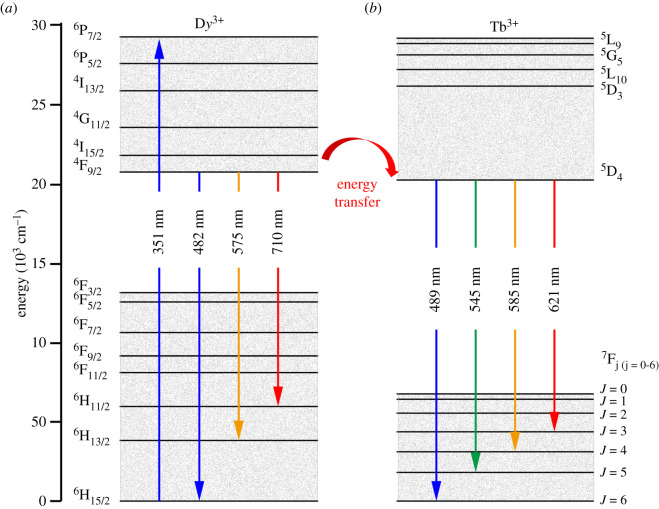
Schematic energy level diagram: (*a*) D*y*^3+^ and (*b*) Tb^3+^ ions for the PL process analysis in the co-doped Ba_1.3_Ca_0.7-*x*-*y*_SiO_4_:*x*D*y*^3+^/*y*Tb^3+^ (*x* = 0.03 and *y* *=* 0.01–0.05) phosphors.

The indispensable condition for energy transfer is the overlap between the emission spectrum of the sensitizer and the excitation spectrum of the activator. Here, the two single-doped (D*y*^3+^ or Tb^3+^) phosphors exhibit the spectral overlap between the broad emission at 482 nm (D*y*^3+^:^4^F_9/2_ → ^6^H_15/2_) and at 489 nm (Tb^3+^:^7^F_6_ → ^5^D_4_) (recall figure 4). Therefore, the resonance type energy transfer from D*y*^3+^ to Tb^3+^ is expected in the co-doped Ba_1.3_Ca_0.7-*x*-*y*_SiO_4_:*x*D*y*^3+^/*y*Tb^3+^ phosphors. To analyse the PL processes in the co-doped phosphors, the schematic energy level diagram was constructed in figure 6. If D*y*^3+^ ions are excited at 351 nm, the excited electrons could be non-radiatively relaxed to the bottom of the conduction band, i.e. ^4^F_9/2_ state via the other lower energy levels by the assistance of phonons [49]. The excited electrons in the conduction band of D*y*^3+^ may be relaxed through the two potential mechanisms. First, the electrons in the ^4^F_9/2_ level of D*y*^3+^ are rapidly relaxed to the H orbitals (^6^H_15/2_, ^6^H_13/2_ and ^6^H_11/2_) through the radiative relaxation process [50], generating the emission light at 482 nm (^4^F_9/2_ → ^6^H_15/2_), 575 nm (^4^F_9/2_ → ^6^H_13/2_) and 710 nm (^4^F_9/2_ → ^6^H_11/2_), respectively (figure 6*a*). Second, the other mechanism is through the energy transfer from the ^4^F_9/2_ energy level of D*y*^3+^ to the adjacent ^5^D_4_ level of Tb^3+^ when an appropriate photon acts as a bridging particle (electromagnetic wave) as shown in figure 6. Here, this energy transfer is mostly irreversible due to energetic reasons, i.e. the state of ^4^F_9/2_ (D*y*^3+^) is higher than that of ^5^D_4_ (Tb^3+^) [14]. In the case of Tb^3+^ ions, the PL is observed at 489 nm (^5^D_4_ → ^7^F_6_), 545 nm (^5^D_4_ → ^7^F_5_), 585 nm (^5^D_4_ → ^7^F_4_) and 621 nm (^5^D_4_ → ^7^F_3_), respectively, as shown in figure 6*b*.

**Figure 7.**
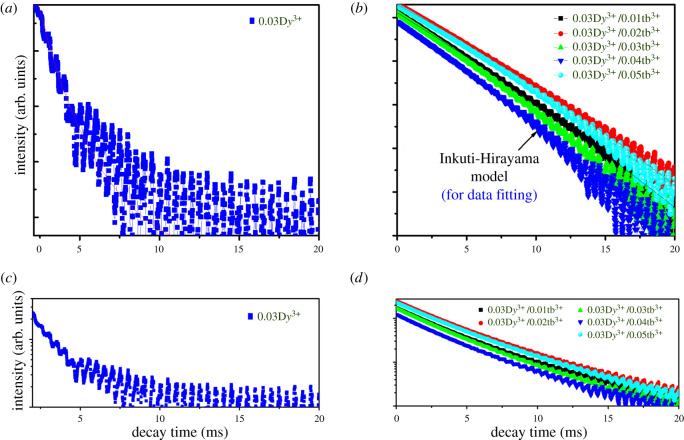
PL decay curves: (*a*) the single-doped Ba_1.3_Ca_0.7-*x*-*y*_SiO_4_:*x*D*y*^3+^/*y*Tb^3+^ (*x* = 0.03 and *y* *=* 0.00) phosphors and (*b*) the co-doped Ba_1.3_Ca_0.7-*x*-*y*_SiO_4_:*x*D*y*^3+^/*y*Tb^3+^ (*x* = 0.03 and *y* *=* 0.01–0.05) phosphors. The *y*-axis with a linear scale in (*a*) and (*b*) was converted to that with a logarithmic scale in (*c*) and (*d*), respectively.

The PL lifetime at 575 nm was measured to investigate the occurrence of energy transfer between D*y*^3+^ and Tb^3+^ ions, in which the excitation and monitoring wavelengths are 351 and 575 nm, respectively. Figure 7 shows the emission decay curves of the single-doped Ba_1.3_Ca_0.7-*x*-*y*_SiO_4_:*x*D*y*^3+^/*y*Tb^3+^ (*x* = 0.03 and *y* *=* 0.00) phosphor and the co-doped Ba_1.3_Ca_0.7-*x*-*y*_SiO_4_:*x*D*y*^3+^/*y*Tb^3+^ (*x* = 0.03 and *y* *=* 0.01–0.05) phosphors. Here, the decay transition of excited electrons is from ^4^F_9/2_ to ^6^H_13/2_ state. Importantly, for the single-doped 0.03D*y*^3+^ phosphors, the decay curve exhibits a double exponential nature due to the different sites of D*y*^3+^ ions (figure 7*a*), whereas for the D*y*^3+^/Tb^3+^ co-doped samples, the decay curves displayed a non-exponential nature (figure 7*b*) [46,51]. Hence, the decay of PL [I(t)] could be fitted according to the below equation,

3.3 $$I(t)=I_{0}+A_{1}e^{-t/\tau_{1}}+A_{2}e^{-t/\tau_{2}},$$

where $I_{0}$, $A_{1}$ and $A_{2}$ are simply constants, whereas $\tau_{1}$ and$\tau_{2}$ are decay constants [52]. In addition, the average lifetime ⟨τ⟩ is expressed as $\left(A_{1}\tau_{1}^{2}+A_{2}\tau_{2}^{2}\right)/\left(A_{1}\tau_{1}+A_{2}\tau_{2}\right)$. Here, the elongated decay time for the co-doped phosphors in figure 7*b* suggests an additional relaxation pathway, leading to the non-exponential nature. For understanding the PL processes, the non-exponential decay curves were fitted according to Inkuti and Hirayama's model [44,46],

3.4 $$I(t)=I_{0}\exp\left[-\left(\frac{t}{\tau_{0}}\right)-Q{\left(\frac{t}{\tau_{0}}\right)}^{3/S}\right],$$

where *S* can be 6, 8 and 10 (for dipole–dipole, dipole–quadrupole and quadrupole–quadrupole interaction, respectively), and $\tau_{0}$ is the intrinsic decay time of donors (D*y*^3+^ ions) in the absence of acceptors (Tb^3+^ ions). The energy transfer parameter (Q) could be expressed as below,

3.5 $$Q=\frac{4\pi }{3}\Gamma \left(1-\frac{3}{S}\right)N_{A}R_{0}^{3},$$

where *S* is the type of interaction and $R_{0}$ is the critical transfer distance defined as the donor–acceptor separation (here, the rate of energy transfer to the acceptors is equal to the rate of intrinsic decay of the donors). Furthermore, the donor–acceptor coupling constant ($C_{DA}$) is defined as $R_{0}^{s}\tau_{0}^{-1}$. The decay lifetime of Ba_1.3_Ca_0.7-*x*-*y*_SiO_4_:*x*D*y*^3+^/*y*Tb^3+^ (*x* = 0.03 and *y* *=* 0.00) was measured to be 2.1 ms, whereas the average decay time of the co-doped Ba_1.3_Ca_0.7-*x*-y_SiO_4_:*x*D*y*^3+^/*y*Tb^3+^ (*x* = 0.03 and *y* *=* 0.01–0.05) phosphors is 3.1 ± 0.2 ms (table 2). Hence, the elongated decay time (approx. 1 ms = 3.1 − 2.1) should originate from the D*y*^3+^/Tb^3+^ energy transfer and/or the microstructural change of samples (e.g. see the crystallite size in table 1). Importantly, because the crystallite size between the single-doped phosphor ($D_{S}$ = 35.86 nm) and the co-doped ones ($D_{S}$ = 36.32 ± 4.0 nm on average) are somewhat similar (recall table 1), the energy transfer process should be the main reason for this elongated PL decay time. However, if we focus on the co-doped samples only, the largest crystallite sample Ba_1.3_Ca_0.7-*x*-*y*_SiO_4_:*x*D*y*^3+^/*y*Tb^3+^ (*x* = 0.03 and *y* *=* 0.01) showed the best PL performance ($\tau_{m}$ ∼ 3.4 ms), indicating that the minimized defects should be helpful for reducing a non-radiative recombination at the defect sites (e.g. the crystallite–crystallite boundaries).

**Figure 8.**
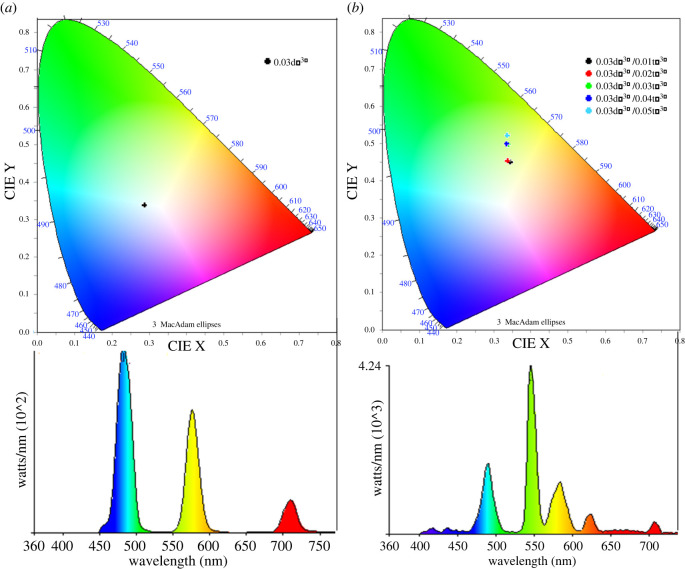
Calculated CIE colour coordinates for emission in (*a*) the single-doped Ba_1.3_Ca_0.7-*x*-*y*_SiO_4_:*x*D*y*^3+^/*y*Tb^3+^ (*x* = 0.03 and *y =* 0.00) and (*b*) the co-doped Ba_1.3_Ca_0.7-*x*-*y*_SiO_4_:*x*D*y*^3+^/*y*Tb^3+^ (*x* = 0.03 and *y* *=* 0.01–0.05) phosphors.

In order to obtain the PL colour of the co-doped phosphor materials, the CIE (i.e. *the International Commission on Illumination*) chromaticity coordinates were obtained from the measured emission spectra. The CIE coordinates of the single- and co-doped phosphors are presented in figure 8. Importantly, the 0.03D*y*^3+^ single-doped phosphor shows the CIE chromaticity coordinates at the white light region (figure 8*a*), whereas the 0.03D*y*^3+^/*x*Tb^3+^ (*x* = 0.01–0.05) co-doped ones display it at the green region very close to the white light location (figure 8*b* and table 2). Hence, the aforementioned phosphor materials could be applied to the white or green lighting devices such as LED or laser [1].

## 4. Conclusion

The single-doped (D*y*^3+^ or Tb^3+^) and co-doped (D*y*^3+^/Tb^3+^) silicate-based ceramic phosphors were synthesized using a gel-combustion method. The average crystallite size ($D_{S}$) of all the hexagonal phosphor samples was approximately 36 nm. However, through the Tb^3+^ co-doping processes, the $D_{S}$ value ranged from approximately 34 to 43 nm, directly affecting the optoelectronic properties (e.g. the larger $D_{S}$, the longer the PL lifetime in co-doped samples). Then, using the SEM-EDX instrument, the morphological and elemental analyses were carried out, showing a dramatic change as a function of the co-dopant Tb^3+^ concentration. Here, in agreement with the XRD data exhibiting the largest crystallite size (approx. 43 nm) for the Ba_1.3_Ca_0.7-*x*-*y*_SiO_4_:*x*D*y*^3+^/*y*Tb^3+^ (*x* = 0.03 and *y* *=* 0.01) sample, the same phosphor material displayed the largest polycrystalline domains. Then, the PL processes were investigated through the PL emission and decay time. Resultantly, the co-doped phosphors with $D_{S}$ ∼ 43 nm exhibited the elongated decay time (approx. 3 ms) based on both the ordered structure and energy transfer process. Finally, the 0.03D*y*^3+^ single-doped phosphor demonstrates the CIE chromaticity coordinate (0.288, 0.338) at the white region, whereas the 0.03D*y*^3+^/0.01Tb^3+^ co-doped one does it (0.346, 0.449) at the green close to the white light coordinate, indicating that the synthesized phosphors could be applied for white or green LED/laser materials.

## Data Availability

Raw data (XRD, PL) and supporting information (EDX, SEM and CIE data) are available from the Dryad Digital Repository: https://doi.org/10.5061/dryad.vq83bk3v3 [53].
